# A Case of Huge Cutaneous Verrucous Carcinoma of the Neck

**DOI:** 10.7759/cureus.15162

**Published:** 2021-05-22

**Authors:** Yusuke Ito, Hironobu Nishijima, Seiji Kishimoto

**Affiliations:** 1 Department of Otorhinolaryngology and Head and Neck Surgery, Kameda Medical Center, Chiba, JPN; 2 Department of Head and Neck Surgery, National Cancer Center Hospital East, Chiba, JPN; 3 Department of Otolaryngology and Head and Neck Surgery, Graduate School of Medicine, The University of Tokyo, Tokyo, JPN

**Keywords:** cutaneous cancer, cutaneous verrucous carcinoma, neck dissection, lymph node metastasis, huge tumor

## Abstract

Verrucous carcinoma (VC) is a rare subtype of squamous cell carcinoma. VC commonly occurs in the mucosa, but rarely occurs in the skin. The treatment for VC is surgical removal of the tumor. Because lymph node metastasis of VC is rare, the indications for prophylactic neck dissection for cutaneous VC of the neck are controversial. Here, we present the case of a 68-year-old man with a huge cutaneous VC of the neck and the long-term clinical course. The tumor occupied the entire right cervical skin, with suspected lymph node metastasis in the affected neck. Tumor resection and neck lymph node dissection were performed. Pathological examination revealed cutaneous VC with invasion to the adjacent tissues and no lymph node metastasis. Cutaneous VC of the neck is likely to grow locally without regional lymph node metastasis regardless of the long-term course and the size of the tumor.

## Introduction

Verrucous carcinoma (VC) is a rare subtype of squamous cell carcinoma (SCC) [1]. VC generally arises from the mucosa rather than from the skin [2]. There are few reports of cutaneous VC of the neck. VC is characterized by local growth and an extremely low incidence of metastasis [2,3]. We report the case of a huge cutaneous VC of the neck with suspected lymph node metastasis, for which we performed tumor excision and elective neck dissection.

## Case presentation

**Figure 1:**
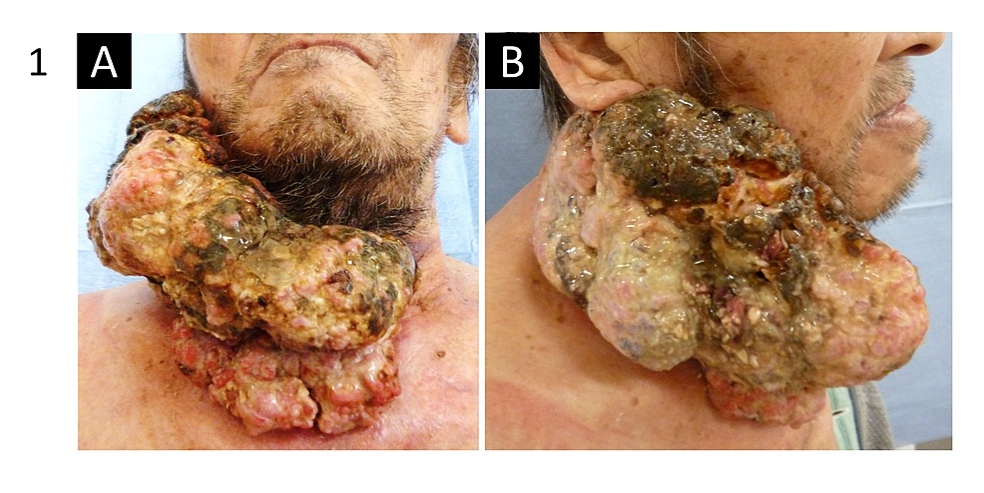
Images of the preoperative patient from the front (A) and the right (B) sides. A large papillary tumor covered the entire right side of the neck.

**Figure 2:**
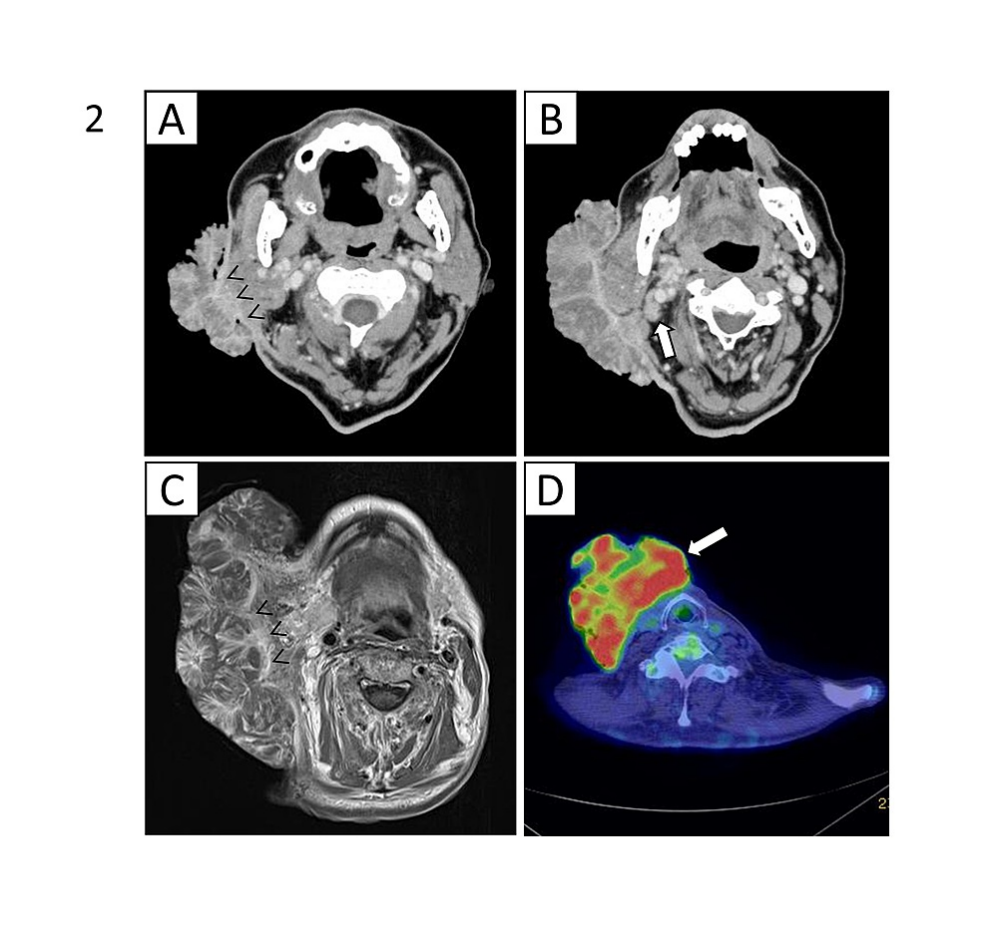
Representative images of contrast-enhanced computed tomography (A, B), T1-weighted gadolinium-enhanced magnetic resonance imaging (C), and positron emission tomography/computed tomography (D). (A) The tumor appeared to invade the parotid gland (arrow head). (B) Enlarged lymph nodes beneath the tumor (arrow). (C) The tumor appeared to invade the sternocleidomastoid muscle (arrow head). (D) A high accumulation (maximum standardized uptake value of 8.88) of 18F-fluoro-2-deoxyglucose was observed in the cutaneous tumor (arrow).

A 68-year-old Asian man with untreated type 2 diabetes visited our department, complaining of a giant right cervical mass, which had been growing gradually for over 10 years. The mass occupied the entire right cervical skin, presenting as a cauliflower-like tumor and measuring approximately 170 mm in diameter (Figure 1). The tumor was bleeding and exhibited infection with an offensive smell. Computed tomography (CT) examination showed a dendritically enhanced 76-mm-thick tumor with suspected infiltration to the right parotid gland (Figure 2A). CT also revealed several enlarged lymph nodes of up to 18 mm beneath the tumor (Figure 2B). Magnetic resonance imaging showed suspected tumor invasion to the sternocleidomastoid muscle (Figure 2C). 18F-fluoro-2-deoxyglucose (FDG) positron emission tomography showed a high accumulation of FDG (maximum standardized uptake value of 8.88) in the tumor without abnormal accumulation in the cervical lymph nodes and distant regions (Figure 2D). Pathological examination of the biopsy specimens of the tumor suggested that the tumor was a well-differentiated SCC and could be VC.

**Figure 3:**
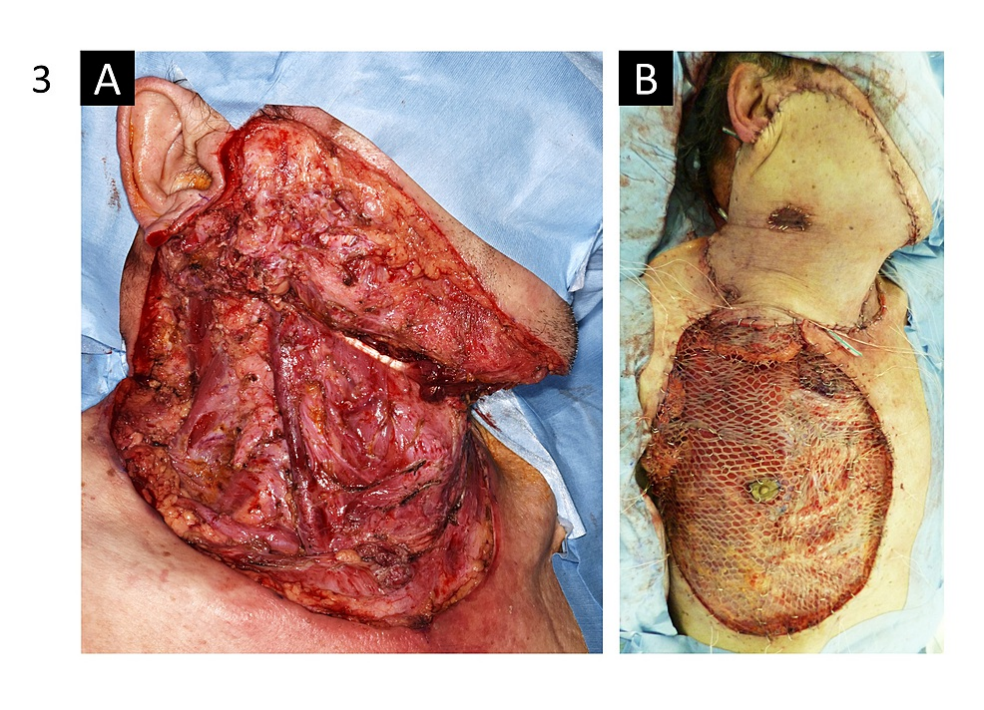
Surgical images. (A) Photograph taken after tumor resection and neck dissection. (B) Photograph taken after reconstruction with a pectoralis major myocutaneous flap and grafting.

We diagnosed the tumor as cutaneous SCC of the neck that had infiltrated into the parotid and sternocleidomastoid muscles, with suspected lymph node metastasis. We performed tumor resection and right neck lymph node dissection (levels I-V), followed by reconstruction with a pectoralis major myocutaneous flap and grafting (Figure 3).

**Figure 4:**
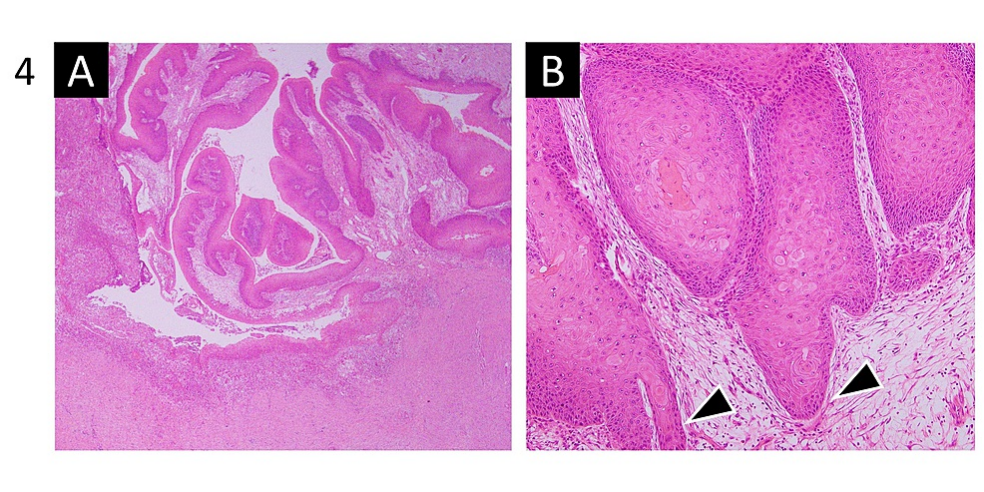
Pathological photomicrograph. (A) Thickened epidermis proliferating, with a papillary surface (magnification, ×20). (B) Well-differentiated squamous rete ridge extending into the underlying tissue (arrow head) (magnification, ×100).

Upon pathological examination, the resected tumor was diagnosed as VC based on the observation that a well-differentiated squamous rete ridge appeared to push into the underlying tissue and no major cytologic atypia could be found within the tumor (Figure 4). Microscopic examination revealed invasion of the tumor into subcutaneous tissues and the platysma but not into the parotid gland or the sternocleidomastoid muscle. The surgical margins of the tumor were negative. Pathological examination also revealed no metastasis in 14 lymph nodes excised from the affected neck. The postoperative course was favorable, there was no further treatment, and no recurrence was observed for nine months after the operation.

## Discussion

VC is a rare subtype of SCC [1] and generally arises from the mucosa rather than from the skin [2]. It typically originates from lesions of the oral cavity, genital area, or plantar surface, but rarely from a cervical skin lesion [4].

VC typically grows slowly, and has been classified as a low-grade malignancy with a favorable prognosis [2,3]. However, because it is an invasive tumor [2,3], VC sometimes destroys adjacent tissues including the bone and cartilage [5]. It is known as a locally expanding tumor with an extremely low incidence of metastasis [2,3].

VC is treated by surgical removal of the tumor [1]. Regarding cutaneous VC of the neck, prophylactic neck dissection is controversial. Few reports are available on cervical lymph node metastasis of VC in the head and neck region. Ferlito and Recher reported a case of lower lip VC with submandibular lymph node metastasis [6]. They also investigated 77 cases of laryngeal VC and found no cervical metastasis, suggesting that neck dissection is unnecessary in VC cases [6]. Kang et al. also stated that elective neck dissection is unnecessary for oral VC [7].

In the present case, the tumor was huge, measuring approximately 170 mm in diameter and 76 mm in thickness. In addition, imaging showed suspected metastasis of the lymph nodes beneath the tumor, and invasion of the tumor to the adjacent tissues. Because histologic evaluation of the lymph node before surgery was difficult, we performed tumor resection and regional lymph node dissection simultaneously. Histologic evaluation revealed no lymph node metastasis, and for the excised enlarged lymph nodes, a diagnosis of reactive swelling caused by infection was made. Concurrent infection is common in VC, producing enlarged lymph nodes that may be mistaken for metastasis [1].

## Conclusions

We reported a case of huge cutaneous VC of the neck with a long-term clinical course. In this case, cervical lymph node metastasis was suspected, and neck lymph node dissection was performed simultaneously with tumor resection. However, histological examination showed no lymph node metastasis. Regardless of the tumor size and the long-term course, cutaneous VC of the neck may grow locally without metastasis.
